# Urinary Biochemistry in the Diagnosis of Acute Kidney Injury

**DOI:** 10.1155/2018/4907024

**Published:** 2018-06-12

**Authors:** Camila Lima, Etienne Macedo

**Affiliations:** ^1^Internal Medicine Department, Nephrology Division, University of Sao Paulo, Sao Paulo, SP, Brazil; ^2^Department of Medicine, Nephrology Division, University of California San Diego, San Diego, CA, USA

## Abstract

Acute kidney injury (AKI) is a common complication, impacting short- and long-term patient outcomes. Although the application of the classification systems for AKI has improved diagnosis, early clinical recognition of AKI is still challenging, as increments in serum creatinine may be late and low urine output is not always present. The role of urinary biochemistry has remained unclear, especially in critically ill patients. Differentiating between a transient and persistent acute kidney injury is of great need in clinical practice, and despite studies questioning their application in clinical practice, biochemistry indices continue to be used while we wait for a novel early injury biomarker. An ideal marker would provide more detailed information about the type, intensity, and location of the injury. In this review, we will discuss factors affecting the fractional excretion of sodium (FeNa) and fractional excretion of urea (FeU). We believe that the frequent assessment of urinary biochemistry and microscopy can be useful in evaluating the likelihood of AKI reversibility. The availability of early injury biomarkers could help guide clinical interventions.

## 1. Introduction

Despite considerable improvement in acute kidney injury (AKI) recognition achieved over the last decades, early diagnosis is still a challenge. The Dialysis Quality Initiative (ADQI) sought a uniform definition of AKI, and the most recent consensus published in 2012 by Kidney Disease Improving Global Outcomes (KDIGO) is currently widely applied in clinical and research scenarios. However, AKI is still fundamentally dependent on changes in serum creatinine and urine output, and both have several limitations [1]. The importance of the timing of diagnosis has been poorly appreciated in AKI, as therapeutic interventions have been lacking and have failed to improve outcomes in many clinical studies. Still, the importance of determining potential reversibility has been emphasized in the early biomarker era. Urinary biochemistry has been utilized since the 1970s, mainly to help differentiate two conditions, namely, reversible (prerenal) or transient AKI (T-AKI), from an established AKI, acute tubular necrosis (ATN), or persistent AKI (P-AKI) [2].

Clinicians usually follow daily serum creatinine concentrations to assess the glomerular filtration rate (GFR), as serum creatinine is freely filtered in the glomerulus, and a small proportion is normally secreted along the tubule [3]. However, serum creatinine levels are influenced by other factors not related to GFR: age, gender, muscle mass, muscle metabolism, medication, and hydration status [4]. Additionally, acute changes in GFR are not accompanied by concomitant increases in serum creatinine, as the balance between production and elimination takes days to occur. Thus, serum creatinine underestimates the degree of loss of renal function, especially in the first 48 hours after the insult [5, 6]. Another common limitation of creatinine is related to the cumulative fluid balance, common in severely ill patients. An increase in the volume of distribution of serum creatinine can cause an underestimation of the creatinine concentration, further delaying the diagnosis [7]. Also, in sepsis, the most frequent cause of AKI in hospitalized patients, production of serum creatinine is decreased [8]. Even in the absence of muscle mass loss, muscular production of serum creatinine has been shown to be reduced in sepsis, further diminishing its value as a marker of AKI [9].

Urinary output also has limitations. First, it is not possible to quantify urine volume in all patients. In children and neonates, the assessment of urine output using diapers is challenging, as weighing diapers is not ideal and subject to errors [10]. In addition, the risk of nosocomial urinary tract infection has reduced the use of urinary catheters and is restricted to severely ill patients [11]. Most importantly, 33% of patients at AKI diagnosis and between 25% and 80% of all cases of AKI are nonoliguric [12–15]. The nonoliguric state may be present in all types of AKI, including those following surgery, trauma, hypotension, nephrotoxins, and rhabdomyolysis. Several factors may contribute to the development of nonoliguric AKI: volume expansion, high-dose potent diuretic agents, and renal vasodilators. Other contributory factors are aggressive fluid resuscitation and improved supportive management of critically ill patients [16]. Therefore, although the residual level of GFR is the primary determinant of urine volume in patients with AKI, there is a distinction between spontaneous and induced urine flow, and urine flow does not correlate with the degree of renal dysfunction.

In some scenarios, urine volume decline may not represent a decline in renal function but an expected response to decreasing renal perfusion. Dehydration in the setting of diarrhea or vomiting is a frequent clinical situation in which renal hypoperfusion can occur, even in the presence of normal blood pressure [17]. In hepatorenal syndrome type 1, considered a reversible disease, the reduction in splanchnic and total vascular resistance occurs as a consequence of increased nitric oxide and endothelium-derived relaxing factor. Any additional insult caused by gastrointestinal losses, bleeding, or therapy with a diuretic or a nonsteroidal anti-inflammatory drug precipitates a further decline in GFR. In this context, it is expected that an increase in renal perfusion can rapidly reverse the decline in GFR or the prerenal state. However, not all hypoperfusion states will respond to fluid expansion. In acute heart failure, impaired cardiac output causes decreased glomerular perfusion pressure and increased venous pressure, reducing the glomerular filtration. Medications that further decrease the effective volume, such as diuretics, or ones that interfere with glomerular perfusion pressure, for example, angiotensin blockers or NSAIDs, often affect the renal autoregulatory response and can trigger prerenal states.

Since the 1940s, laboratory tests to distinguish T-AKI conditions from P-AKI have been used. These diagnostic parameters present various exceptions, and the distinction between prerenal and renal causes are frequently not accurate. The plasma (P) urea/creatinine ratio, urine (U) osmolality, U/P osmolality, U/P creatinine ratio, urinary Na^+^ level, and fractional excretions of Na^+^ (FeNa) are the most frequently used tests. Serum U/P creatinine ratio helps to identify whether the oliguria is a result of water reabsorption (U/Pcr > 20) or loss of tubular function (U/Pcr < 20). In reversible states, the reabsorption of sodium is increased, not only from the increase in proximal tubular reabsorption of water but also by the increase in aldosterone level secondary to hypovolemia. The concentrations of sodium (UNa) and chloride (UCl) in the urine have been known to be high during established phases of P-AKI. Although the accuracy of UNa alone in determining the cause of AKI is limited, the renal failure index (UNa ÷ U/P creatinine) or the fractional excretion of sodium (FeNa or U/PNa ÷ U/P creatinine × 100) was found to have a high degree of accuracy in differentiating between TAKI and PAKI. Despite these caveats, the tests are easy to perform, cheap, noninvasive, and available in daily clinical practice. Understanding the limitations of urinary biochemistry can reveal their potential benefits to assist in the diagnosis, differentiation, and management of AKI [18, 19].

## 2. Fractional Excretion of Sodium (FeNa)

Espinel [20] conducted a landmark study with the fractional excretion of sodium (FeNa), which was one of the first analyses of urinary biochemistry used to differentiate T-AKI from P-AKI. The interpretation of FeNa is based on the premise that intact tubules reabsorb sodium in the prerenal states while the injured tubules do not [20, 21]. In that study published in 1976, FeNa was evaluated in 17 patients in the oliguric phase of acute renal failure. In patients who recovered from AKI, FeNa was less than 1% (Table 1), and in those with P-AKI, FeNa was more than 3% (*p* < 0.01) [20]. Other studies have confirmed FeNa as a possible tool to differentiate functional and structural AKI. Miller et al. [22] and Espinel and Gregory [23] showed that FeNa is more than 1% in oliguric and nonoliguric ATN and urinary tract obstruction, and low (<1%) in prerenal azotemia and in acute glomerulonephritis.

Despite the small number of patients, these studies revealed the potential to distinguish T-AKI from P-AKI. Prerenal AKI is accepted as a reversible form of renal dysfunction, caused by factors that compromise renal perfusion. The term has been used as part of a dynamic process that begins with a reversible condition, prerenal state, and can progress to an established disease, ATN. Although we consider P-AKI an established form of cell injury, most likely representing acute tubular necrosis, in most studies and in clinical practice the histopathological diagnosis is uncertain. The confirmation is most often retrospective, once renal function recovers over days to weeks.

The concept of T-AKI occurs when FeNa less than 1% is indicated, as increased reabsorption of sodium is the appropriate response of functioning nephrons to reduced renal perfusion; values greater than 1% are consistent with P-AKI due to inappropriate sodium excretion in the setting of tubular damage. Nevertheless, several subsequent studies were unable to reproduce these findings. Saha et al. [24] evaluated FeNa in 54 patients with acute intrinsic renal failure in whom renal biopsy was performed: 48 had tubulointerstitial nephritis and 6 had acute glomerulonephrites. By dividing patients into three groups based on FeNa < 1%, >1%, and >3%, they found no association between histological findings and FeNa [24]. Bagshaw et al. evaluated the use of urinary biochemistry in septic AKI [25]. They found lower urinary sodium (UNa) in septic-AKI patients, as compared to non-septic-AKI patients, but the index was not useful to differentiate prerenal failure from P-AKI in these patients [18, 26]. The physiopathological mechanisms involved in septic AKI are different from those in ischemic-associated AKI [27]. The inflammatory and hormone system activation results in arterial vasodilatation and induction of an increase in tubular sodium reabsorption and a decrease in urinary sodium concentration. Thus, FeNa in this setting could be an inadequate parameter to evaluate the hypoperfusion state.

The primary goal in differentiating functional from structural AKI has been the assessment of reversibility with fluid therapy. The concept of prerenal implies that hemodynamic improvement can increase renal blood flow and reverse the prerenal state. Thus, these indices have been used to differentiate T-AKI from P-AKI. This differentiation could avoid inappropriate fluid infusions in patients who have P-AKI [28], possibly decreasing the risk of pulmonary edema and mechanical ventilation, both risk factors for increased mortality [29, 30]. On the other hand, low FeNa does not always imply functional AKI that can be reversible with fluid expansion. Especially in septic patients, this difference is unclear, as studies have shown a low value of FeNa to be common in septic patients with P-AKI. Vanmassenhove et al. [28], in a prospective study, analyzed FeNa and other biomarkers in 107 sepsis patients at admission on an intensive care unit (ICU), 4 hours and 24 hours after admission. They showed lower levels of FeNa in no AKI versus T-AKI; however, FeNa < 1% was found in 77.3% of all cohorts, and 50% of patients had a value < 0.36%, considered as the reference value in their study. Another recent study carried out by Bagshaw et al. [25] found similar results with FeNa < 1% in 57% of the cohort. These results emphasize the need for a revision of the reference value in septic patients.

Two other points deserve emphasis concerning the use of urinary biochemistries as a diagnostic tool in AKI. In some patients with nonoliguric ATN, P-AKI, and some vascular/glomerular disorders (acute glomerulonephritis, vasculitis, and thrombotic thrombocytopenic purpura) early in the course of urinary tract obstruction, urinary chemical indices can be indistinguishable from those seen with prerenal AKI and T-AKI. Conversely, several acute renal parenchymal disorders (e.g., interstitial nephritis, severe ischemic nephropathy, and exacerbations of chronic renal failure) are found to have a low value of FeNa, despite their severity and likelihood of reversibility [31–33].

In patients with normal kidney function, the level of FeNa depends on several factors, such as glomerular filtration and sodium intake. Thus, a single cutoff value may not be adequate in the interpretation of FeNa as a marker of tubular function. A recent study analyzed FeNa, GRF, and sodium intake in 761 children without AKI [34]. The authors compared the difference between measured and predicted FeNa based on urinary sodium excretion and creatinine clearance and showed that the predicted FeNa was significantly lower than the measured FeNa in the children with tubular dysfunction. This study highlights the effect of salt intake and eGFR in FeNa values. Another common caveat in the interpretation of FeNa is the use of diuretics. Diuretic use is a common practice in AKI patients with volume overload. Diuretics decrease sodium reabsorption, increasing FeNa and so interfering with the performance of FeNa in differentiating between T-AKI and P-AKI [35]. In a prospective study [36] with 99 patients, 64 of whom received diuretics, FeNa values were not different within the 43 patients with T-AKI and 21 with P-AKI.

## 3. Fractional Excretion of Urea (FeU)

Fractional excretion of urea nitrogen (FeU) (Table 1) may be a more useful tool than FeNa in the differential diagnosis of AKI. Urea reabsorption is primarily dependent on passive forces and is, therefore, less influenced by diuretic therapy [18, 37]. FeU relates inversely to the proximal reabsorption of water, and urea reabsorption leads to a decrease in FeU and an increase in the BUN/creatinine ratio. In a prospective study, Carvounis et al. found that FeU < 35% (Table 1) was associated with a 98% chance of prerenal failure [37]. FeU had a high sensitivity (85%), a high specificity (92%), and a high positive predictive value, being a useful tool to differentiate T-AKI from P-AKI. Similarly, Dewitte et al. concluded that a FeU of less than 40% was a sensitive and specific index for differentiating T-AKI from P-AKI [38].

Still, there are also some limitations in the use of FeU. In osmotic diuresis and with the use of mannitol or acetazolamide, the proximal tubular reabsorption of salt and water is impaired, so there can be an increase in FeU, even in states of hypoperfusion [39]. The same can occur when a patient is given a high protein diet or presents excessive catabolism. In septic patients, the release of cytokines can interfere with the urea transporters in the kidney and colon; in these patients, FeU is not a good indicator of reversibility [40]. In critically ill patients, the use of FeU has also been questioned. In a multicenter study carried by Wlodzimirow et al. [41], 150 critically ill patients evaluated the performance of FeU to differentiating transient T-AKI in 51 patients from P-AKI in 41 patients. The use of FeU had a reduced ability to discriminate T-AKI from P-AKI on the day of diagnosis of AKI (AUC 0.61) and on the first (AUC 0.61) and second (AUC 0.58) days prior AKI. Other similar results were found in multicenter studies in an ICU population, reported by Darmon et al. [42] and Pons et al. [43]. Both studies concluded that FeU was not helpful in differentiating T-AKI from P-AKI on admission to ICU. Despite these discouraging findings, other studies have proposed the use of a combination of both FeNa and FeU tests, which may increase diagnostic sensitivity and specificity in the differential diagnosis of AKI, especially in the context of patient history, physical examination, and urinalysis [44].

## 4. Urine Microscopy

Urine microscopy (UM) is a frequently forgotten tool to evaluate AKI. The distinction between T-AKI and P-AKI is classically made by the presence of granular casts and muddy-brown or mixed cellular casts [45–49]. However, the number of renal tubular epithelial cell casts can be also helpful to distinguish less severe ATN and P-AKI cases and predict the likelihood of T-AKI. Marcussen et al. [50], in 51 patients with AKI, showed that the numbers of cylinders were higher in patients with ATN. In addition, 12 patients who needed dialysis had granular, waxy, leukocytic, and broad-cast cylinders. Chawla et al. [51] revisited this issue and developed a new score with the objective of standardizing urinary sediment analysis. This score was tested in 30 patients, 18 with ATN. They were able to show an association of higher scores with lower rates of renal recovery. In 2009, Perazella et al. [52] proposed a urinary sediment scale (Table 2) to evaluate the differential diagnosis of AKI. They created three groups, according to the numbers of granular casts and renal tubular epithelial cells (RTEC) present. Zero granular cast and zero RTEC were more frequent in the prerenal state, while ATN and P-AKI patients had higher numbers of granular casts and RTEC. More recently, the revision of Perazella and Coca [21] suggests that the urinary sediment scale used in association with early biomarkers can be helpful in the prognosis of AKI severity, the need for RRT, and prevention of mortality.

In sepsis-associated AKI, UM has been shown to be more useful than urinary biochemistry, as it is less affected by hydration status and medications. Bagshaw et al. [53], evaluating the UM of 83 critical patients, showed that it was effective in discriminating septic AKI from nonseptic, with worse sediment found in septic AKI, and it was associated with worsening AKI, the need for RRT, and mortality. In a prospective study, Schinstock et al. [54] analyzed the use of NGAL and UM to detect AKI in patients admitted from the emergency department. Urinary NGAL levels had an only fair sensitivity (65%) and specificity (65%) to differentiate no AKI versus stages 1, 2, or 3 (area under the curve 0.70). In that study, the urinalysis with microscopy was very specific (91%) but not very sensitive (22%), with an area under the curve of 0.57.

These findings reveal the possible use of urinary biochemistry and UM in the differential diagnoses of AKI. The majority of studies of spot chemistries have been performed at a single point of time, relatively late in the course of AKI. The lack of serial data is an essential factor to consider in the interpretation of these results, as AKI is a dynamic process. Following the progression of daily urinary biochemistry and UM can improve the value of these parameters in AKI differential diagnosis. During the early phases of AKI, the renal tubular function is intact. Later, cell injury may result in the loss of tubular cell polarity. The resulting urine chemistries, therefore, are dependent on the phase of the course in which they were obtained.

## 5. Biomarkers

Over the last decades, several early markers of kidney injury have been proposed: urinary cystatin C [55], urinary Kidney Injury Molecule-1 (KIM-1) [56], urinary interleukin 18 (IL-18) [57], and urinary/plasma neutrophil gelatinase-associated lipocalin (NGAL) [58]. Although evaluated in different settings, few biomarkers have been introduced to clinical practice. Several studies have demonstrated the value of NGAL for early AKI diagnosis [59] and prediction of severity and need for dialysis [60]. Recently, biomarkers of cell cycle arrest, insulin-like growth factor-binding protein 7 (IGFBP7), and tissue inhibitor of metalloproteinases-2 (TIMP-2) [61] have added mechanistic insight into AKI physiopathology and increased hope for the prospect of early diagnosis and interventions for AKI. The availability of rapid assays for some of these markers [62, 63] and several ongoing trials will soon provide more guidance on early AKI management. However, the increased cost and lack of evidence of improvement in patients' hard outcomes continue to be the main limitations for their use.

## 6. Conclusion

We believe that, by monitoring the parameters of urinary biochemistry and UM, we can predict the likelihood of early AKI recovery. The combined assessment of the urinary biochemistries of FeNa and FeU and UM can facilitate the differential diagnosis of AKI. Large studies with an early diagnosis of AKI should include urinary biochemistry/microscopy and correlate with early biomarkers of injury to help in the recognition of reversibility.

## Figures and Tables

**Table 1:** Urinary index equations and reference values.

Urinary indices	Formula	Traditional reference value
FeNa	FeNa = [(Nau/NaS)/(cru/crP)] × 100	<1% transient AKI >1% persistent AKI
FeU	FeU = [(Uu/US)/(cru/crP)] × 100	<35% transient AKI >35% persistent AKI

Na—sodium, cr—creatinine, u—urinary, S—serum, P—plasma, U—urea, and AKI—acute kidney injury. Modified from [20, 37].

**Table 2:** Differential diagnosis of AKI based on the numbers of casts in the urinary sediment.

Kinds of casts	Numbers	Reference values
Granular casts	0	Transient AKI
	1 to 10 by LPF	Persistent AKI
Epithelial cell casts	0	Transient AKI
	1 or more by HPF	Persistent AKI

AKI (acute kidney injury), LPF (low-power field), and HPF (high-power field). Modified from [51–53].
